# The national food safety initiative.

**DOI:** 10.3201/eid0402.980239

**Published:** 1998

**Authors:** S Binder, R Khabbaz, B Swaminathan, R Tauxe, M Potter


### The National Food Safety Initiative

For many years, the Centers for Disease Control and Prevention (CDC) and state and local health departments have investigated and controlled outbreaks of foodborne diseases and conducted limited passive surveillance for specific foodborne pathogens. However, recent changes in the food supply and in the consuming public have changed the type and range of infections caused by food and have made traditional approaches to disease surveillance increasingly inadequate. In particular, new technology which facilitates the national and international distribution of food to a population of consumers increasingly vulnerable to infection by new microorganisms has changed the character of foodborne disease in the United States in ways that demand more and better information for decision making.

### Foodborne Disease Surveillance

In 1997, President Clinton announced an administration initiative to create a nationwide early warning system for foodborne diseases, enhanced food safety inspection, and expanded food safety research, training, and education. The National Food Safety Initiative will meet the challenges presented by the changing nature of our food safety concerns by strengthening and broadening surveillance and response systems at CDC and the state and local health departments. The following areas of national surveillance for foodborne diseases are addressed by the initiative: rapid identification of and effective response to foodborne hazards; identification of large, diffuse outbreaks that have a low attack rate within one health jurisdiction but affect the health of many people within a larger geographic area; and evaluation of the nature and scope of food safety problems. In addition, the National Food Safety Initiative will 1) link CDC, state and local health departments, and other federal agencies within a powerful electronic network developed by CDC for rapid sharing of microbial subtyping information, including the digitalized images of subtyping patterns needed for molecular epidemiology, and 2) will enhance and expand the activities of FoodNet, the interagency foodborne disease component of CDC's emerging infectious disease active surveillance program to improve the quantitative base for foodborne disease.

### Strengthening and Broadening the Base of Surveillance

Under the National Food Safety Initiative, CDC is building epidemiologic and laboratory capacity in state and local health departments so they can quickly and effectively identify foodborne hazards, investigate and control outbreaks, and develop effective programs to prevent additional illnesses from the same source. Routine surveillance and outbreak response, critical to the proper functioning of health departments, have been neglected at the state and local levels because of limited resources and competing priorities. Therefore, CDC is providing training and equipment needed by the health departments to update surveillance systems and diagnostic capability for bacterial, parasitic, and viral foodborne diseases. In addition, CDC, state epidemiologists, and state public health laboratory directors are working to define the elements of core capacity for effective response to food safety problems. The results of this evaluation will provide guideposts for efficient allocation of initiative resources in 1999 and beyond to construct a truly effective national early warning system for foodborne diseases.

CDC's response to foodborne hepatitis A virus infections, supported in 1998 by the National Food Safety Initiative, illustrates the value of better surveillance. Existing hepatitis A surveillance data document food as a vehicle of transmission in only 2% to 5% of cases. On the basis of these data, economic analysis suggests that routine immunization of food workers to prevent foodborne transmission of hepatitis A virus (with a vaccine already available) is not a cost-effective nationwide public health response. However, existing surveillance data document a source of exposure (food or otherwise) for only half of the reported cases of hepatitis A. Therefore, CDC is collaborating with health departments in three states to more fully describe the epidemiology of hepatitis A infections for which routine investigation fails to determine a source. In addition, CDC will evaluate the effectiveness of the algorithm used to determine which patrons of food service establishments with infected food workers will receive immune globulin for postexposure prophylaxis and will establish a genetic sequence-based library of hepatitis A virus strains circulating in the United States and other countries to permit the effective use of molecular epidemiology in investigations of hepatitis A outbreaks and sporadic cases.

Diarrheal disease results in large numbers of physician visits and hospitalizations each year in the United States. However, the infectious cause of the illness is frequently not determined, which prevents good surveillance and impedes development of effective prevention strategies. Therefore, CDC is embarking on studies of mild and severe diarrhea to determine to the greatest extent possible the bacterial, parasitic, and viral agents involved. Similarly, applied research at CDC that is improving diagnostic and subtyping methods for parasitic foodborne diseases and caliciviruses will support future surveillance and outbreak response for these foodborne pathogens, as well as help define the extent of their contribution as causes of sporadic foodborne disease.

### Establishing Electronic Networks and Generating Quantitative Data

Another aspect of CDC's response to foodborne disease under the National Food Safety Initiative is the development of an electronic network that links public health laboratories for rapid data sharing, including the digitalized images of pulsed-field gel electrophoresis (PFGE) patterns. These patterns can be used to distinguish related cases that may be part of an outbreak from background disease so that risk factors can be identified and control measures can be established. By the end of 1997, CDC had laboratories in 16 state health departments trained and equipped to perform PFGE subtyping of *Escherichia coli* serotype O157:H7 with standardized techniques so the results could be compared among laboratories. Microbiologists in federal regulatory laboratories were also trained in these standardized techniques so *E. coli* O157:H7 isolated from food and animal samples could be directly compared with clinical isolates from human cases. Laboratories in four states were established as regional resources to make PFGE subtyping available to surrounding states, and one was directly linked to CDC's PFGE library of strains so it could query CDC's database in addition to being able to submit electronic data for analysis. The other three regional laboratories and laboratories in USDA and FDA will be directly linked to CDC's database early in 1998; the remaining six FoodNet sites will be linked later in the year. In addition, in 1998 six more states will be trained and equipped to perform PFGE subtyping of *E. coli* O157:H7, training will be provided to health department laboratories in two foreign countries, PFGE subtyping of *Salmonella* Typhimurium will begin, and methods for subtyping *Salmonella* Enteritidis and other foodborne pathogens will be developed.

Changes in food safety regulations (e.g., regulatory use of Hazard Analysis and Critical Control Points systems and international requirements for quantitative risk assessment) and increasing emphasis on the cost-effectiveness of prevention programs require epidemiologic data on foodborne disease that cannot be provided by routine passive surveillance systems based on laboratory and physician diagnoses. In response, CDC collaborated with U.S. Department of Agriculgure, Federal Drug Administration, and the five state health departments enrolled in CDC's emerging infections active surveillance program in 1995 to create FoodNet. FoodNet expands the quantitative database on foodborne diseases, expedites response to certain food safety concerns, and provides a test bed for evaluating new prevention strategies. FoodNet activities include 1) comprehensive documentation of foodborne infections through active laboratory-based surveillance, 2) determination of health and food-handling practices of the population under surveillance, 3) determination of health-care practices by surveys of the physicians and diagnostic laboratories in the surveillance areas, and 4) implication of specific foods as vehicles for various foodborne pathogens by conducting case-control studies of sporadic infections.

These efforts have shown that *Campylobacter* is the most commonly diagnosed cause of diarrheal disease, that great geographic variations in the frequency of *E. coli* O157:H7 infections and other foodborne pathogens suggest a need for targeted control measures, and that approximately 1.4 diarrheal illness episodes occur per person per year in the United States.

FoodNet began with active surveillance of sporadic infections with seven bacterial pathogens in the five sites. In 1997, two new sites were added, and in 1998 an eighth active surveillance site will be added; active surveillance for *Cryptosporidium*, *Cyclospora*, and *Calicivirus* infections will be included, and intensive investigation of foodborne disease outbreaks will be initiated. Many bacterial pathogens, including those that are foodborne, are becoming resistant to antibiotics. To measure this change in resistance, CDC, in collaboration with FDA, USDA, and the states, began monitoring resistance levels of several foodborne bacteria. This information is being used to promote the prudent use of antibiotics in human health and in agriculture.

### Other Food Safety Initiative Projects

In 1999, in addition to continuing and extending surveillance activities, CDC plans to support the following projects through the National Food Safety Initiative: school-based health education to teach foodborne disease prevention to a new generation of consumers; a foodborne disease diagnosis and surveillance component to CDC's Field Epidemiology Training Program, which helps Ministries of Health in foreign countries (many of which are major food exporters to the United States) improve their surveillance and diagnostic capabilities; and new methods of determining the risk for exposure to pesticide residues in food. The National Food Safety Initiative is building the public health framework for an effective and efficient response when novel foods from new areas that contain unfamiliar foodborne hazards are introduced into the marketplace.

**Sue Binder, Rima Khabbaz, Bala Swaminathan, Robert Tauxe, and Morris Potter**
Centers for Disease Control and Prevention,
Atlanta, Georgia, USA

### Meeting Summary

### Conference on Global Disease Elimination and Eradication as Public Health Strategies, February 26, 1998

The goals of the conference were to assess the role of elimination and eradication in decreasing global disease and in using health resources more effectively. Two hundred invited representatives from 81 organizations and 34 countries participated in the multidisciplinary conference; the proceedings will be published in late 1998 in a supplement to the Bulletin of the World Health Organization.

The working definitions during the conference were those developed at the Dahlem Workshop on the Eradication of Infectious Diseases, March 1997. Disease control: reduction of disease incidence, prevalence, illness, or death to a locally acceptable level as a result of deliberate efforts; continued intervention measures are required to maintain the reduction. Disease elimination: reduction to zero of the incidence of a specific disease in a defined geographic area as a result of deliberate efforts; continued intervention measures are required. Disease eradication: permanent reduction to zero of the worldwide incidence of infection caused by a specific agent as a result of deliberate efforts; intervention measures are no longer needed.

The successful smallpox program and the ongoing poliomyelitis and dracunculiasis (Guinea worm disease) programs served as models in the discussions on eradication. The malaria, yellow fever, and yaws programs of earlier years were recognized as unsuccessful but to have contributed to better understanding of the biologic, social, political, and financial complexities and responsibilities of disease eradication. The conference addressed five major areas: sustainable development, noncommunicable diseases, bacterial diseases, parasitic diseases, and viral diseases. The following is a summary of the conclusions.

#### Sustainable Development

Eradication programs should have two objectives: 1) eradication of the disease and 2) strengthening and further development of health systems, especially functions such as monitoring and surveillance, supervision, and program management. Potential risks of eradication to the health system and health development include the diversion of resources from basic services and other priorities in countries where the disease being eradicated is perceived to be of lower priority. An additional concern is the failure to accurately estimate the human and financial needs of the eradication efforts. Potential benefits for health development should be identified and delineated at the start of any eradication initiative. Measurable targets should be set, and the program should be held accountable for achieving them. Resources for

